# Epidemiology and risk factors of methicillin-resistant *Staphylococcus aureus* and vancomycin-resistant *enterococci* infections in Zhejiang China from 2015 to 2017

**DOI:** 10.1186/s13756-019-0539-x

**Published:** 2019-05-30

**Authors:** Lin Huang, Rong Zhang, Yanyan Hu, Hongwei Zhou, Junmin Cao, Huoyang Lv, Shi Chen, Shibiao Ding, Gongxiang Chen

**Affiliations:** 1 Department of Clinical Microbiology, The Second Affiliated Hospital of Zhejiang University School of Medicine, 88 Jiefang Road, Hangzhou, Zhejiang 310009 People’s Republic of China; 2 Zhejiang Provincial Hospital of TCM, Hangzhou, 310006 Zhejiang China; 3 Centre of Laboratory Medicine, Zhejiang Provincial People’s Hospital, People’s Hospital of Hangzhou Medical College, Hangzhou, 310014 Zhejiang China; 4 Hangzhou Third Hospital, Hangzhou, 310009 Zhejiang China; 5 Hangzhou Red Cross Hospital, Hangzhou, 310003 Zhejiang China

**Keywords:** Epidemiology, Risk factor, Methicillin-resistant *Staphylococcus aureus*, Vancomycin-resistant *enterococci*

## Abstract

**Background:**

Gram-positive bacteria are dangerous and challenging agents of infection due to their increasing resistance to antibiotics. We aim to analyse the epidemiology and risk factors of methicillin-resistant *Staphylococcus aureus* (MRSA) and vancomycin-resistant *enterococci* (VRE) in Zhejiang China.

**Methods:**

Gram-positive bacteria (including *S. aureus*, *Enterococcus faecalis* and *Enterococcus faecium*) were collected from eighty-six hospitals of eleven cities in Zhejiang China from 2015 to 2017. The detection rates of MRSA and VRE infection were calculated for the non-duplicated isolate according to year, region, hospital level, patient age, specimen type and patient category. Meanwhile, the detected resistances of MRSA, *E. faecalis* and *E. faecium* to different antibiotics from 2015 to 2017 were compared. The risk factors and the differences in MRSA and VRE detection rates were compared using odds ratio (OR) with 95% confidence interval (95% CI) and Chi-square test respectively.

**Results:**

From 2015 to 2017, the detection rates of MRSA and VRE decreased gradually. The cities with the highest MRSA and VRE detection rates tended to be adjacent; for example, the neighbouring cities Hangzhou and Quzhou had simultaneously high rates of MRSA and VRE infection. Patients from IIIA hospital who were older than 75 years and in the intensive care unit (ICU) were most at risk. No vancomycin-resistant isolate was found in MRSA. Resistance of *E. faecalis* and *E. faecium* to vancomycin and linezolid decreased slightly and then maintained a low level.

**Conclusions:**

The detection rates of MRSA and VRE stayed at moderate and low levels during the three year period of this study, while local dissemination was found in MRSA and VRE isolates. Sustained surveillance is necessary to prevent the spread or clonal dissemination of drug-resistant strains in Zhejiang China.

## Background

Gram-positive bacteria, such as *Staphylococcus aureus, Enterococcus faecalis,* and *Enterococcus faecium* are dangerous and challenging agents of infection due to their increasing resistance to antibiotics [1]. Among these, *S. aureus* are the most frequently isolated, making up 29.1% of the isolated Gram-positive bacterial populations, and infections caused by *S. aureus* (both community-associated or nosocomial-associated) are reported all over the world [2, 3]. *Enterococci* (including *E. faecalis* and *E. faecium*) are the second frequently isolated Gram-positive bacteria at 19.5%. Past generations of *enterococci* were mainly associated to urinary-tract infection, but recently more and more *enterococci* are isolated from other infections [4], and an outbreak of vancomycin resistant *enterococci* (VRE) in a solid organ transplant unit was reported in 2018 [5].

The epidemiology of resistance in Gram-positive bacteria has undergone major changes in recent decades, with methicillin-resistant *S. aureus* (MRSA) and VRE now being of international concern [6]. Antimicrobial resistances of Gram-positive bacteria have been reported in many countries [7–9]; however, it was only in recent years that *vanM*-carrying *E. faecalis* strains were isolated from patients and the clinical environment in the Second Affiliated Hospital of Zhejiang University School of Medicine [10], we speculate that if *vanM* infection has been spreading in the Zhejiang province, it may lead to an increase of VRE overall. In China, although several local studies have examined the prevalence of resistance in Gram-positive bacteria [11, 12], the studies usually only cover the tertiary hospitals. Therefore, the present study is a retrospective surveillance covering a wider range of hospitals, including tertiary and secondary hospitals. Our aim is to seek trends in antimicrobial resistance among clinical isolates of important Gram-positive bacteria in the Zhejiang province of China.

## Materials and methods

### Bacterial isolates

We extracted data of *S. aureus*, *E. faecalis* and *E. faecium* infections from the Zhejiang surveillance system’s outpatient and inpatient records from January 1, 2015 to December 31, 2017. The number of surveyed hospitals for each city was as follows: Hangzhou (*n* = 24), Huzhou (*n* = 4), Jiaxing (*n* = 11), Jinhua (*n* = 9), Lishui (*n* = 6), Ningbo (*n* = 10), Quzhou (*n* = 5), Shaoxing (*n* = 6), Taizhou (*n* = 5), Wenzhou (*n* = 5), and Zhoushan (*n* = 1). Sample collection was in accordance with the clinical microbiology manual [13]. To avoid duplicate isolates, only one isolate from the same species was included per patient, as determined by the personal identifying code and hospital name. Species identification of the isolates was performed by standard biochemical methods, automated system (the Vitek 2 compact, BD Phoenix-100, MicroScan WalkAway-96) or Matrix-Assisted Laser Desorption/Ionization Time of Flight Mass Spectrometry (MALDI-TOF-MS).

### Antimicrobial susceptibility testing

Standard operation procedures were established according to the Clinical and Laboratory Standards Institute’s (CLSI) criteria, M100-S24 [14]. Every participating laboratory conducted the antibiotic susceptibility testing for the clinical isolates using the Kirby-Bauer disk diffusion method or the commercialized automated system following the instrument specifications. *S. aureus* (ATCC 25923, ATCC 43300) and *E. faecalis* (ATCC 29212, ATCC 51299) were used as quality control strains for the testing.

### Statistical analysis

We analysed the risk factors for MRSA and VRE proportions, and for the multivariable model, year, region, hospital level, patient age, specimen type and patient category were considered. The lowest detection rates of MRSA and VRE were set as contrasts. The risk factors and the differences in MRSA and VRE detection rates were compared using odds ratio (OR) with 95% confidence interval (95% CI) and Chi-square test. Chi-square values were corrected when the quantities of VRE isolates were less than forty. Results with *P*-value < 0.05 were considered statistically significant. SPSS 19.0 (IBM Company, Chicago, IL) and WHONET 5.5 (WHO) software were used for all statistical analyses.

## Results

### Distribution of MRSA, *Enterococci* and VRE isolates

During the years 2015–2017, the total number of MRSA isolates increased from 9292 to 10,237 cases; in contrast, VRE isolates decreased from 173 to 137 cases (data not shown). Among the 11 cities that participated in the survey, Hangzhou contributed 37.6% of all MRSA isolates (29,866 cases); and 41.0% of all *Enterococci* isolates (58,329 cases), although the population of Hangzhou is only about 9.5 millon (16.5% of the Zhejiang province population). This high number of isolates may be due to Hangzhou having more IIIA hospitals, which were the contributors of 67% of all MRSA and *enterococci* isolates. However, Hangzhou also contributed 245 VRE isolates, which was 54.2% of all of the VRE isolates in the study, and a much higher percentage than the province’s MRSA and *enterococci* contributions. MRSA were most frequently isolated from respiratory samples (14,339 or 48.0% of all samples); VRE were dominantly isolated from urine samples (313 or 69.2% of all samples) (data not shown). The number of samples based on age of patient and inpatient vs. out-patient were also categorized. Patients older than 75 and patients who were hospitalized (especially those in the ICU) had dominantly higher MRSA, *enterococci* and VRE isolation rates compared to other patient groups (data not shown).

### Resistance rates of MRSA, *E. faecalis* and *E. faecium* to antimicrobial agents from 2015 to 2017

**Fig. 1:**
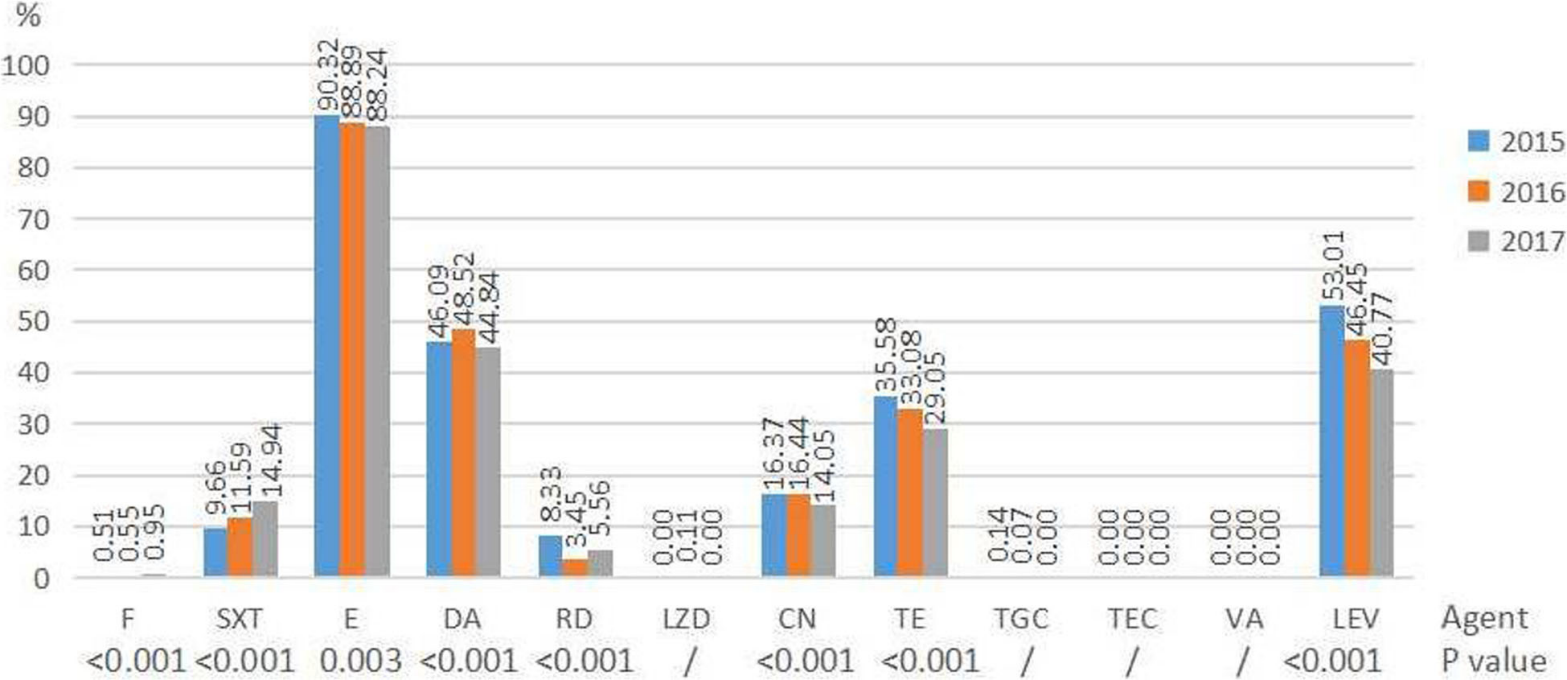
Resistance rates of MRSA to antimicrobial agents during 2015 to 2017. / No comparison when appearing resistance rate was 0%. Results of nitrofurantoin were only from urine isolates. Abbreviations: F nitrofurantoin, SXT trimethoprim-sulfamethoxazole, E erythromycin, DA clindamycin, RD rifampicin, LZD linezolid, CN gentamicin, TE tetracycline, TGC tigecycline, TEC teicoplanin, VA vancomycin, LEV levofloxacin

During the period of 2015 to 2017, the resistance rates of MRSA to trimethoprim-sulfamethoxazole and nitrofurantoin increased from 9.66 to 14.94% (*P* < 0.001) and from 0.51 to 0.95% (*P* < 0.001) respectively. The resistance rates of MRSA to erythromycin, tetracycline, tigecycline and levofloxacin decreased from 90.32 to 88.24% (*P* = 0.003), from 35.58 to 29.05% (*P* < 0.001), from 0.14% to 0, and from 53.01 to 40.77% (*P* < 0.001) respectively (Fig. 1). MRSA showed 100% susceptibility to vancomycin and teicoplanin, however, a few linezolid-resistant strains were found in 2016. The resistance rates of *E. faecium* to nitrofurantoin and gentamicin-high level increased from 51.90 to 55.47% (*P* < 0.001) and from 34.15 to 41.67% (*P* < 0.001) increased respectively. The resistance rates of *E. faecium* to linezolid, tetracycline, teicoplanin and vancomycin decreased from 1.15 to 0.67% (*p* = 0.168), from 38.33 to 30.77% (*p* = 0.243), from 0.51 to 0.16% (*P* < 0.001), and from 1.69 to 0.90% (*p* = 0.006) respectively (Table 1). The resistance rate of *E. faecalis* to gentamicin-high level increased from 7.32 to 41.67% (*P* < 0.001). The resistance rates of *E. faecalis* to ampicillin, nitrofurantoin, linezolid, tetracycline, and levofloxacin decreased from 5.72 to 3.04% (*p* = 0.021), from 3.95 to 2.56% (*P* < 0.001), from 3.17 to 2.73% (*p* = 0.002), from 77.29 to 76.32% (*P* < 0.001), and from 20.78 to 19.79% (*p* = 0.009) respectively (Table 1). Tigecycline showed 100% susceptibility against *E. faecalis* and *E. faecium*. The resistance rates of *E. faecium* to ampicillin, nitrofurantoin, ciprofloxacin, gentamicin-high level, levofloxacin, teicoplanin and vancomycin were higher than that of *E. faecalis*, while lower resistance rates to tetracycline and linezolid were seen in *E. faecium* in comparison to *E. faecalis*.

**Table 1:** Resistance rates (%) of *E. faecalis* (E.fa) and *E. faecium* (E.fm) to antimicrobial agents during 2015 to 2017

Antimicrobial agent	2015		2016		2017		*P* value	
	E.fa(*n* = 9109)	E.fm (*n* = 7106)	E.fa(*n* = 10,740)	E.fm(*n* = 9135)	E.fa (*n* = 11,860)	E.fm (*n* = 10,379)	E.fa	E.fm
Ampicillin	5.72	88.22	4.66	89.58	3.04	89.13	0.021	< 0.001
Nitrofurantoin	3.95	51.90	3.19	54.96	2.56	55.47	< 0.001	< 0.001
Gentamicin-High	7.32	34.15	18.06	41.57	41.67	41.67	< 0.001	< 0.001
Ciprofloxacin	22.54	89.13	23.14	90.47	22.05	90.32	0.009	0.147
Linezolid	3.17	1.15	2.97	1.00	2.73	0.67	0.002	0.168
Tetracycline	77.29	38.33	76.90	33.75	76.32	30.77	< 0.001	0.243
Tigecycline	0	0	0	0	0	0	/	/
Teicoplanin	0	0.51	0	0.45	0	0.16	/	< 0.001
Vancomycin	0.67	1.69	0.37	1.22	0.42	0.90	< 0.001	0.006
Levofloxacin	20.78	87.49	20.43	89.04	19.79	88.47	0.009	0.195

/ No comparison when appearing resistance rate was 0%

### Resistance rates of *S. aureus* and *Enterococci* to antimicrobial agents among different patients

**Fig. 2:**
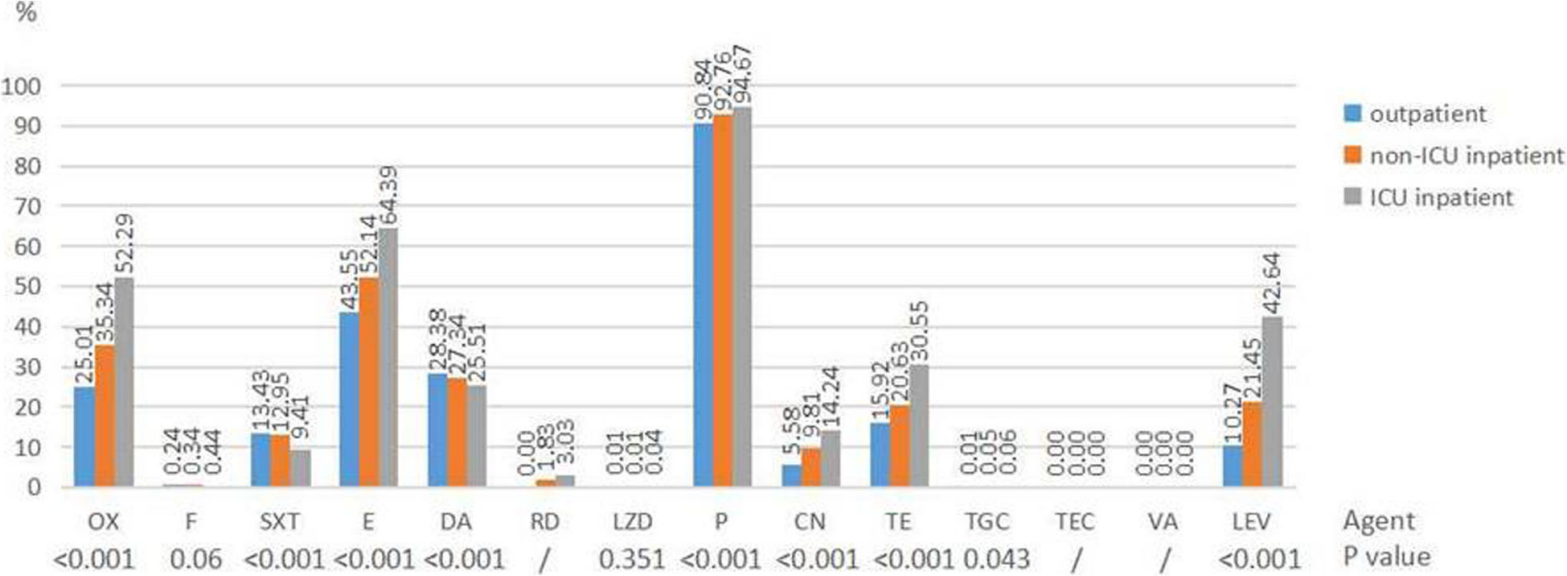
Resistance rates of *S. aureus* to antimicrobial agents among different patients. / No comparison when appearing resistance rate was 0%. Results of nitrofurantoin were only from urine isolates. Abbreviations: OX oxacillin, F nitrofurantoin, SXT trimethoprim-sulfamethoxazole, E erythromycin, DA clindamycin, RD rifampicin, LZD linezolid, P penicillin, CN gentamicin, TE tetracycline, TGC tigecycline, TEC teicoplanin, VA vancomycin, LEV levofloxacin

**Fig. 3:**
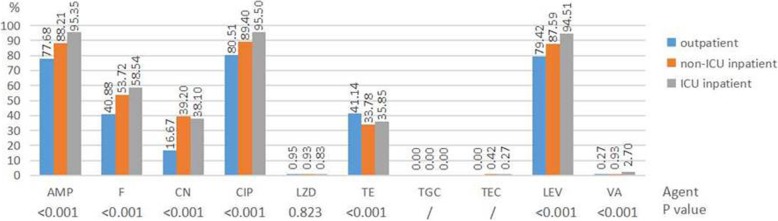
Resistance rates of *enterococci* (*E. faecalis* and *E. faecium*) to antimicrobial agents among different patients. / No comparison when appearing resistance rate was 0%. Results of nitrofurantoin, tetracycline, ciprofloxacin and levofloxacin were only from urine isolates. Abbreviations: AMP ampicillin, F nitrofurantoin, CN gentamicin-high level, CIP ciprofloxacin, LZD linezolid, TE tetracycline, TGC tigecycline, TEC teicoplanin, LEV levofloxacin, VA vancomycin

For isolates collected from the outpatients, non-ICU inpatients and ICU inpatients, resistance rates of *S. aureus* to all the antimicrobial agents increased gradually except for clindamycin and trimethoprim-sulfamethoxazole (Fig. 2). Among which, the resistance rates to oxacillin, gentamicin and levofloxacin increased more dramatically. Resistance rates of *enterococcus* (including *E. faecalis* and *E. faecium*) to all the antimicrobial agents increased gradually except for linezolid and tetracycline (Fig. 3).

### Analysis of risk factors associated with MRSA and VRE strains

**Fig. 4:**
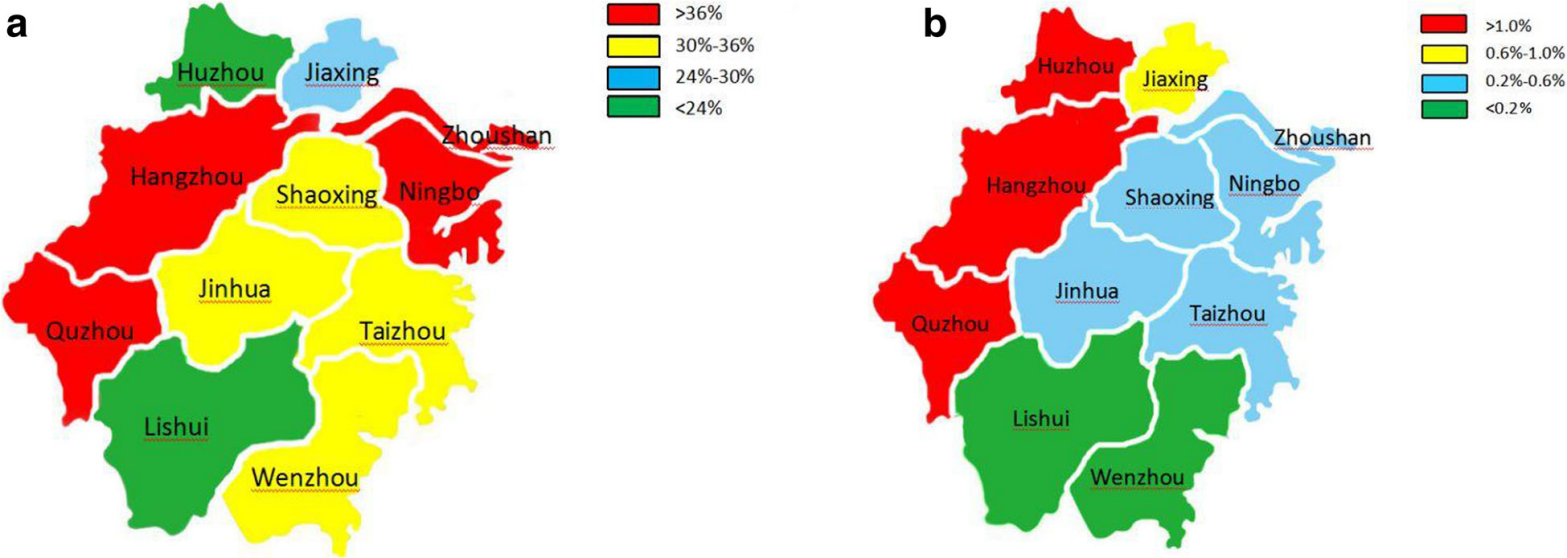
Distribution of MRSA (**a**) and VRE (**b**) by geographic area

In multivariable analysis for risk factors associated with MRSA and VRE (Table 2), we observed that the 2015 isolation rates of MRSA (OR = 1.022, 95% CI 1.010–1.035, *p* < 0.001) and of VRE (OR = 1.005, 95% CI 1.003–1.006, *p* < 0.001) were higher than those in 2017. Compared to Huzhou, Zhoushan was the most likely city to have MRSA isolates (OR = 1.775, 95% CI 1.676–1.880, *p* < 0.001). Compared to Lishui, Huzhou was the most likely city to have VRE isolates (OR = 1.025, 95% CI 1.017–1.033, *p* < 0.001). The cities with high detection rates of MRSA were distributed in the east and west of the Zhejiang province, and the cities with high detection rates of VRE were distributed in the west of the Zhejiang province (Fig. 4). Isolates from tertiary hospitals were more likely to be MRSA and VRE than isolates from secondary hospitals. Analysis based on age groups revealed that isolates derived from patients older than 75 years had the highest proportion of MRSA (OR = 1.443, 95% CI 1.409–1.478, *p* < 0.001) and VRE (OR = 1.011, 95% CI 1.009–1.013, *p* < 0.001). Isolates from bile had the highest proportion of MRSA (OR = 1.385, 95% CI 1.151–1.665, *p* < 0.001) and isolates from blood had the highest proportion of VRE (OR = 1.007, 95% CI 1.003–1.010, *p* < 0.001). Patients who were in ICU had the highest proportion of MRSA (OR = 1.439, 95% CI 1.407–1.473, *p* < 0.001) and VRE (OR = 1.019, 95% CI 1.013–1.025, *p* < 0.001).

**Table 2:** Analysis of risk factors associated with MRSA and VRE strains

	MRSA		VRE	
	OR (95%CI)	*P* value	OR (95%CI)	*P* value
Year				
2015	1.022 (1.010–1.035)	< 0.001	1.005 (1.003–1.006)	< 0.001
2016	1.018 (1.006–1.030)	0.002	1.001 (0.999–1.003)	0.214
2017	1	–	1	–
Region				
Hangzhou	1.329 (1.295–1.363)	< 0.001	1.010 (1.008–1.012)	< 0.001
Huzhou	1	–	1.025 (1.017–1.033)	< 0.001
Jiaxing	1.109 (1.080–1.139)	< 0.001	1.006 (1.003–1.009)	< 0.001
Jinhua	1.229 (1.195–1.265)	< 0.001	1.005 (1.002–1.008)	0.001
Lishui	1.030 (1.002–1.059)	0.045	1 (0.997–1.003)	1
Ningbo	1.406 (1.365–1.447)	< 0.001	1.002 (1–1.004)	0.054
Quzhou	1.453 (1.398–1.511)	< 0.001	1.017 (1.011–1.024)	< 0.001
Shaoxing	1.189 (1.156–1.224)	< 0.001	1.003 (1.001–1.006)	0.015
Taizhou	1.197 (1.163–1.231)	< 0.001	1.005 (1.002–1.009)	0.001
Wenzhou	1.282 (1.245–1.319)	< 0.001	1	–
Zhoushan	1.775 (1.676–1.880)	< 0.001	1.004 (0.999–1.010)	0.079
Hospital Level				
IIIA (*n* = 46)	1.267 (1.213–1.322)	< 0.001	1.016 (1.009–1.024)	0.031
IIIB (*n* = 24)	1.189 (1.138–1.242)	< 0.001	1.010 (1.002–1.019)	0.143
IIA (*n* = 15)	1.139 (1.089–1.190)	< 0.001	1.016 (1.006–1.025)	0.045
IIB (*n* = 1)	1	–	1	–
Age				
< =7d	1.130 (1.063–1.201)	< 0.001	*	*
8d-28d	1.085 (1.043–1.129)	< 0.001	*	*
1 m-1y	1.054 (1.030–1.079)	< 0.001	*	*
2y-3y	1	–	1.008 (0.998–1.019)	0.015
4y-19y	1.015 (0.992–1.037)	0.203	1	–
20y-43y	1.057 (1.035–1.080)	< 0.001	1.004 (1.002–1.005)	0.044
44y-59y	1.104 (1.080–1.128)	< 0.001	1.007 (1.005–1.009)	0.002
60y-74y	1.190 (1.164–1.217)	< 0.001	1.006 (1.004–1.008)	0.004
75 + y	1.443 (1.409–1.478)	< 0.001	1.011 (1.009–1.013)	< 0.001
Specimen type				
Blood	1.142 (1.101–1.185)	< 0.001	1.007 (1.003–1.010)	< 0.001
Bile	1.385 (1.151–1.665)	< 0.001	1.001 (0.999–1.004)	0.403
Respiratory	1.318 (1.278–1.360)	< 0.001	1.003 (0.995–1.011)	0.515
Urine	1.186 (1.139–1.235)	< 0.001	1.006 (1.004–1.008)	< 0.001
Secretion	1.115 (1.081–1.150)	< 0.001	1	–
Puncture fluid	1	–	1.004 (1–1.009)	0.012
stool	1.342 (1.262–1.426)	< 0.001	1.001 (0.997–1.005)	0.839
Other	1.137 (1.102–1.173)	0.002	1 (0.998–1.002)	1
Patient category				
Outpatient	1	–	1.004 (0.995–1.014)	0.4
Inpatient-non ICU	1.132 (1.118–1.147)	< 0.001	1	–
Inpatient-ICU	1.439 (1.407–1.473)	< 0.001	1.019 (1.013–1.025)	< 0.001

OR odds ratio, CI confidence interval

IIIA number of bed more than 500, comprehensive examination score more than 900 points

IIIB number of bed more than 500, comprehensive examination score between 750 and 899 points

IIA number of bed between 100 and 499, comprehensive examination score more than 900 points

IIB number of bed between 100 and 499, comprehensive examination score between 750 and 899 points

Comprehensive examination including departments, staffing, management level, technical level, work quality and technical facilities

d day old, m month old, y year old

Respiratory containing sputum and bronchoalveolar lavage fluid

Secretion containing pus and wound swab

Puncture fluid containing hydrothorax, ascites, articular cavity fluid, pericardial fluid and Cerebrospinal fluid

* No VRE isolate

## Discussion

It is a global trend that the drug resistance of Gram-positive bacteria decreases gradually. In our study, there is a slight decline in the isolation rates of MRSA (from 34.98 to 33.53%). The downward trend is also observed in France, Germany and the UK [15, 16], but it is not seen in Saudi Arabia, where MRSA is maintaining its high level [17]. The use of alcohol-based hand-rub and decolonization with antimicrobial agents may have helped to reduce MRSA transmission. As with other studies, linezolid, tigecycline and vancomycin are the most active agents against MRSA [18]. There were no MRSA isolates that were resistant to vancomycin and teicoplanin. Unlike other reports, the minimum inhibitory concentration (MIC) of MRSA to vancomycin was distributed around 0.5 and 1 mg/L, and was stable during the three years study [19]. These results may be attributed to vancomycin being an unlikely empirical therapy due to its narrow-spectrum and its injection-only administration, thus resistances may not have had a chance to form. Meanwhile, vancomycin belongs to the highest limit level in the classification management system of antibiotics. Linezolid resistances in coagulase negative *staphylococci* are greater than resistance in *S. aureus* [20, 21]. *Staphylococcus capitis* isolates were the highest in Zhejiang province, and the resistance rates to linezolid increased from 1.8% in 2014 [20] to 3.5% in 2016 [21]. This is due to an outbreak of linezolid-resistant *S. capitis* infection in Zhejiang. Since the first report of linezolid resistance in methicillin-resistant coagulase negative *staphylococci* in the Second Affiliated Hospital of Zhejiang University School of Medicine in 2011, linezolid resistance in human clinical isolates has become an increasing problem in China [22, 23], and the *cfr*-carrying plasmid has appeared in *S. aureus* [24]. We isolated several linezolid-resistant *S. aureus* strains in 2016, but not in 2015 and 2017. No outbreaks of linezolid resistance were seen in *S. aureus* isolates*.*

The results of the present study show that the resistance rates of *E. faecium* are greater than the resistance rates of *E. faecalis,* and the resistance rates of *E. faecium* to ampicillin and quinolones are more than 80%. Therefore infections caused by *E. faecium* present a serious clinical challenge for physicians [25], and treatment options for these infections are limited. Vancomycin is one treatment option that could be considered. In the last ten years, a weak downward trend for VRE cases was found worldwide and may be seen in the Zhejiang province. We also confirmed that the resistance rate of *enterococcus* to vancomycin remains at a low level, and showed downward trends, similar to other reports in China and lower than that in Ireland [26]. We found that the resistance rates of *E. faecalis* and *E. faecium* to vancomycin are higher than the resistance rates to teicoplanin. We speculate that strains with the dominant gene *vanB* may have the highest resistance to vancomycin [27]. These may have a different resistance than strains with the *vanM* gene, seen in China [28] or in strains with *van*A dominance, seen in Poland [29]. In our study, resistance rate of *E. faecalis* to linezolid increased from 1.6% in 2008 to 2.97% in 2016 [21] and linezolid resistance was higher in *E. faecalis* than in *E. faecium.* In the past, mutations in the central loop of domain V of the 23S rRNA represented the most common mechanism of oxazolidinone resistance in *enterococci*, with G2576 T (*Escherichia coli* numbering) as the predominant mutation [30]. Increasingly, transferable oxazolidinone resistance from the multi-resistance genes *cfr* and *optrA* are being reported all over the world [31–33]. Furthermore, it was reported that the *optrA* gene was detected more frequently from food-producing animals than from humans [34].

In the multivariable analysis for risk factors associated with MRSA and VRE, we found that Hangzhou and Quzhou simultaneously had high MRSA and VRE detection rates. As the provincial capital of Zhejiang, Hangzhou has more tertiary hospitals and receives a greater number of critically ill patients from other cities. As a relatively under-developed city, Quzhou may have a poorer sanitary arrangement, and the compliance with antibiotics may be worse. Meanwhile, we found that the cities of relatively high MRSA and VRE detection rates tend to neighbour one another. This phenomenon may be caused by the increased likelihood of interaction between the populations and patients of these adjacent cities, allowing greater dissemination of MRSA and VRE isolates. To be a patient in a IIIA hospital (the highest classified and possibly largest hospital type in China) and a patient in the ICU ward are the greatest risk factors associated with MRSA and VRE infection. These findings are in accordance with the literature [35, 36] and can be attributed to patients with severe co-morbidities. Stratifying the data by patient age, it is observed that the proportion of MRSA and VRE was the highest in isolates from elderly patients older than 75 years, but was the lowest from children aged 2 years - 3 years and infants younger than 1 year. The high numbers of MRSA and VRE isolates from elderly patients may be due to these patients having more underlying diseases and a greater history of antibiotic use than the child and the infant group. As well, decreased nutrition and immune function, often seen in the elderly, may also be contributing elements. Other identified risk factors associated with MRSA and VRE include having a source of isolates. We found that puncture fluid (containing hydrothorax, ascites, articular cavity fluid, pericardial fluid and cerebrospinal fluid) has the lowest proportion (21.32%) of MRSA, though the underlying reason for this phenomenon needs to be further studied. In terms of treatment, we should be wary of methicillin-sensitive *S. aureus* when *S. aureus* is isolated from puncture fluid in Zhejiang province. With the noted exception of blood and urine, there is no difference in the specimen types in proportions of VRE isolates.

These findings will provide valuable information for infection control practices. Although many surveillance projects of antibiotic resistance have been carried out in China, they always cover only the tertiary hospitals. In our study, a wider range of scenarios were seen where resistant strains could occur, indicating the importance of performing regional antibiotic resistance surveillance. The current study had some limitations: we had a relatively short span of time (3 years) for the data collection, we had a limited number of IIB hospitals to contribute data to the study, and because ours was a retrospective analysis, we had an inability to obtain the original strains. In the future, we would like to expand this surveillance to cover more IIB and rural area clinics and also collect the original strains for intensive study.

## Conclusion

The detection rates of MRSA stayed at moderate levels, and VRE stayed at low levels during the last three years, and local dissemination was found in MRSA and VRE isolates. The highest risk factors for MRSA and VRE infection were patient status in a IIIA hospital, age older than 75 years and hospitalization in the ICU ward. As a result of our findings, we suggest that sustained surveillance is necessary to prevent the spread or clonal dissemination of drug-resistant strains in Zhejiang China.

## Abbreviations

CI: Confidence interval
ICU: Intensive care unit
MALDI-TOF-MS: Matrix-Assisted Laser Desorption/ Ionization Time of Flight Mass Spectrometry
MIC: Minimum inhibitory concentration
MRSA: Methicillin-resistant *Staphylococcus aureus*
OR: Odds ratio
VRE: Vancomycin-resistant *enterococci*
